# TDP-43 CSF Concentrations Increase Exponentially with Age in Metropolitan Mexico City Young Urbanites Highly Exposed to PM_2.5_ and Ultrafine Particles and Historically Showing Alzheimer and Parkinson’s Hallmarks. Brain TDP-43 Pathology in MMC Residents Is Associated with High Cisternal CSF TDP-43 Concentrations

**DOI:** 10.3390/toxics10100559

**Published:** 2022-09-24

**Authors:** Lilian Calderón-Garcidueñas, Elijah W. Stommel, Ingolf Lachmann, Katharina Waniek, Chih-Kai Chao, Angélica González-Maciel, Edgar García-Rojas, Ricardo Torres-Jardón, Ricardo Delgado-Chávez, Partha S. Mukherjee

**Affiliations:** 1 College of Health, The University of Montana, Missoula, MT 59812, USA; 2 Universidad del Valle de México, Mexico City 14370, Mexico; 3 Department of Neurology, Geisel School of Medicine at Dartmouth, Hanover, NH 03755, USA; 4 Roboscreen GmbH, 04129 Leipzig, Germany; 5 Instituto Nacional de Pediatría, Mexico City 04530, Mexico; 6 Instituto de Ciencias de la Atmósfera y Cambio Climático, Universidad Nacional Autónoma de México, Mexico City 04510, Mexico; 7 Independent Researcher, Mexico City 04310, Mexico; 8 Interdisciplinary Statistical Research Unit, Indian Statistical Institute, Kolkata 700108, India

**Keywords:** ALS, air pollution, Alzheimer’s, Aβ1–42, α synuclein, children, cerebrospinal fluid, cisternal CSF, fronto-temporal dementia, Metropolitan Mexico City, nanoparticles, olfactory bulb granule cells, PM_2.5_, Parkinson’s, hyperphosphorylated tau, TDP-43

## Abstract

Environmental exposures to fine particulate matter (PM_2.5_) and ultrafine particle matter (UFPM) are associated with overlapping Alzheimer’s, Parkinson’s and TAR DNA-binding protein 43 (TDP-43) hallmark protein pathologies in young Metropolitan Mexico City (MMC) urbanites. We measured CSF concentrations of TDP-43 in 194 urban residents, including 92 MMC children aged 10.2 ± 4.7 y exposed to PM_2.5_ levels above the USEPA annual standard and to high UFPM and 26 low pollution controls (11.5 ± 4.4 y); 43 MMC adults (42.3 ± 15.9 y) and 14 low pollution adult controls (33.1 ± 12.0 y); and 19 amyotrophic lateral sclerosis (ALS) patients (52.4 ± 14.1 y). TDP-43 neuropathology and cisternal CSF data from 20 subjects—15 MMC (41.1 ± 18.9 y) and 5 low pollution controls (46 ± 16.01 y)—were included. CSF TDP-43 exponentially increased with age (*p* < 0.0001) and it was higher for MMC residents. TDP-43 cisternal CSF levels of 572 ± 208 pg/mL in 6/15 MMC autopsy cases forecasted TDP-43 in the olfactory bulb, medulla and pons, reticular formation and motor nuclei neurons. A 16 y old with TDP-43 cisternal levels of 1030 pg/mL exhibited TDP-43 pathology and all 15 MMC autopsy cases exhibited AD and PD hallmarks. Overlapping TDP-43, AD and PD pathologies start in childhood in urbanites with high exposures to PM_2.5_ and UFPM. Early, sustained exposures to PM air pollution represent a high risk for developing brains and MMC UFPM emissions sources ought to be clearly identified, regulated, monitored and controlled. Prevention of deadly neurologic diseases associated with air pollution ought to be a public health priority and preventive medicine is key.

## 1. Introduction

Neurodegenerative disorders with complex environmental and genetic pathogenesis start decades before clinical symptomatology is present [1,2,3]. Quadruple aberrant neural pathology starting in childhood and environmental damage to fetal brains in utero likely have short- and long-term neuropsychiatric and neuropathological outcomes [2,3,4]. Air pollution has been associated with neuroinflammation and oxidative stress, and fine particulate matter (PM_2.5_), ozone (O_3_), and nitrogen dioxide (NO_2_) are strongly associated with higher risks of several types of dementia and Parkinson’s disease (PD) [5,6,7,8,9,10,11]. Psychiatric outcomes, including depression and suicide, are reported in association with air pollution exposures [12,13,14,15]. The pediatric impact of traffic pollution includes cognitive deficits, altered neurobehavioral performance, structural brain changes and increased risk of attention deficit/hyperactivity and autistic spectrum disorders, and maternal exposures to traffic air pollution during late pregnancy contribute to oxidative stress and inflammation in newborn children [16,17,18,19,20,21,22,23]. A key component of PM_2.5_ is the ≤100 nm fraction: ultrafine PM (UFPM) and nanoparticles (NPs). Anthropogenic UFPM are primarily generated via combustion (e.g., vehicular sources) and subsequent processes of particle nucleation, coagulation and vapor condensation, while NPs are frequently referred to as engineered or manufactured because they are designed and generated for a particular purpose; e.g., medical [24]. Anthropogenic UFPM and industrial NPs are ubiquitous and have detrimental neural impacts [25,26,27,28,29].

We have previously shown that cerebrospinal fluid (CSF) concentrations of cytokines and chemokines, cellular prion protein (PrPc), total tau (T-tau), tau phosphorylated at threonine 181 (P-Tau), amyloid Aβ1–42, α-synuclein (t-α-syn and d-α-synuclein), brain-derived neurotrophic factor (BDNF), insulin and leptin can be used to distinguish MMC children from low pollution controls [30,31,32,33]. Moreover, CSF myelin basic protein autoantibodies and nickel concentrations, as well as Mn, Ni and Cr concentrations in frontal tissues, are higher in MMC cases vs. low pollution controls [34,35]. Of particular relevance to this work are the significantly lower amyloid Aβ1-42 and BDNF concentrations in MMC children versus low pollution controls (*p* = 0.005 and 0.02, respectively) and the fact that non-P-Tau cases showed significantly faster increases in MMC residents versus controls (*p* = 0.005) [31,32,33].

The overlap of several aberrant CSF proteins in children and young MMC adults, including hyperphosphorylated tau, beta amyloid, alpha synuclein and TDP 43 [1,2,4], drove our current investigation of MMC children and adults with TDP-43 in CSF and brain tissues versus low pollution controls, and we selected hospital-diagnosed amyotrophic lateral sclerosis (ALS) as an example of patients with a clinical syndrome with complex biological determinants characterized by TDP-43 pathology and high TDP-43 lumbar CSF concentrations. Moreover, our 203 forensic autopsy MMC data cases for residents ≤ 40 y, with 18% showing TDP-43 pathology, drove our interest in the relationship between cisterna magna TDP-43 concentrations and brain pathology.

Two research groups were critical in defining the relationship and the interpretation of protein concentrations in cisternal versus lumbar CSF; the work by Reiber [36,37] and by Peyron et al. [38]. More specifically, Reiber states that “*nonlinearly increasing protein concentrations between ventricular and lumbar CSF are fitting to a Gaussian error function, the differential of the nonlinear concentration distribution function between blood and CSF*” [36] and that the relationship between blood-CSF and blood–brain barrier (BBB) dysfunction is an expression of reduced CSF, or CSF flow rate, and CSF protein gradients [37]. These conclusions support the positive relationship between cisterna magna TDP-43 concentrations and our 3 ± 1.2 h after death autopsy findings [38].

TDP-43 nuclear depletion and aggregation are hallmarks of ALS and frontotemporal dementia (FTD) [39,40,41,42]. TDP-43 immunocytochemical profiles in children and young MMC adults have shown loss of nuclear expression and powdery cytoplasmic particles in the substantia nigrae pars compacta and non-motor neurons, as well as significant involvement of mesencephalic, pontine and medullary reticular formation [2,41,42,43,44]. Of particular concern, we have reported sleep disorders in MMC residents [45] along key brainstem sleep and arousal hubs, showing solid UFPM from anthropogenic combustion, mainly diesel exhaust, as well as non-exhaust sources coming from tire and brake wearing and from engineered NPs [46,47,48]. Although FTD and ALS epidemiological data are not extensively studied in Mexico [49,50], the presence of overlapping quadruple pathologies in MMC children and young adults [2,45,46] raises the question of why such diagnoses are missing in our populations. Providing an early ALS diagnosis is clinically difficult [51], and development of research criteria for the diagnosis of prodromal behavioral variant frontotemporal dementia (bvFTD) is ongoing [52]. Given the current challenges in defining mechanisms underlying the development of TDP-43 pathology and the early identification of individuals at risk for FTD and ALS, we hypothesized that CSF TDP-43 quantification from air pollution-exposed residents is highly relevant. Our rationale is summarized as follows: (i) even at the earliest age examined, CSF TDP-43 shows significant differences from contrasting polluted environments; (ii) the measurement of cisterna magna CSF TDP-43 and its correlative relationship with TDP43 pathology in the brain tissue of children and young adults is crucial; and (iii) The contribution of UFPM and NP exposures to changes in CSF TDP-43 levels in highly exposed residents versus clean air controls supports the hypothesis that UFPM fractions [53,54,55] are highly neurotoxic and lead to TDP-43 pathology.

In sum, the purpose of the present study was to assess, in MMC versus lower pollution age-matched subjects, the impact of lifetime exposures to highly polluted environments on CSF TDP-43 burden. Our results identify lumbar CSF TDP-43 increasing exponentially with age in young urbanites with high exposures to PM pollution and TDP-43 cisternal CSF levels of 572 ± 208 pg/mL forecasting TDP-43 pathology in young MMC residents.

There is a strong association between TAR DNA-binding protein 43, frontotemporal lobar degeneration (FTLD) and amyotrophic lateral sclerosis (ALS) [39,40,41,42,43,44,51,52]; thus, our findings in young, highly PM-exposed megacity residents are highly relevant for the global medical community, from both the neuropathology and clinical viewpoints.

## 2. Materials and Methods

### 2.1. Study Cities and Air Quality

**Figure 1:**
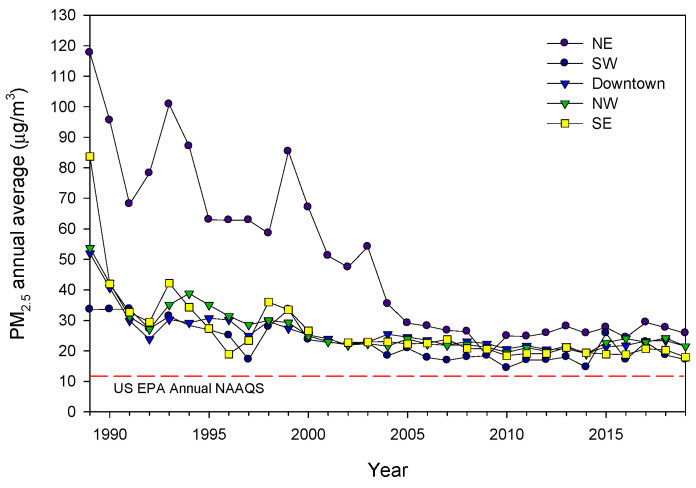
Time series trends showing annual mean 24 h PM_2.5_ concentrations, averaged over 3 years, for five representative MMC monitoring stations from 1990 to April 2020 and their comparison with the respective annual US EPA NAAQS. Annual means from the years before 2004 were estimated from available information of PM_10_ since 1990 and the mean slope of the correlation PM_10_ vs. PM_2.5_ between 2004 and 2007. Data from: http://www.aire.cdmx.gob.mx/default.php# (accessed on 4 June 2022).

MMC residents have been chronically exposed to significant concentrations of fine particulate matter (PM_2.5_) and O_3_ for the last three decades [56,57,58,59]. Figure 1 shows the time series trend of annual mean 24 h PM_2.5_ concentrations, averaged over 3 years, for representative MMC monitoring stations in the period between 1990 to April 2020 and their comparison with PM_2.5_ from the US EPA NAAQS.

**Figure 2:**
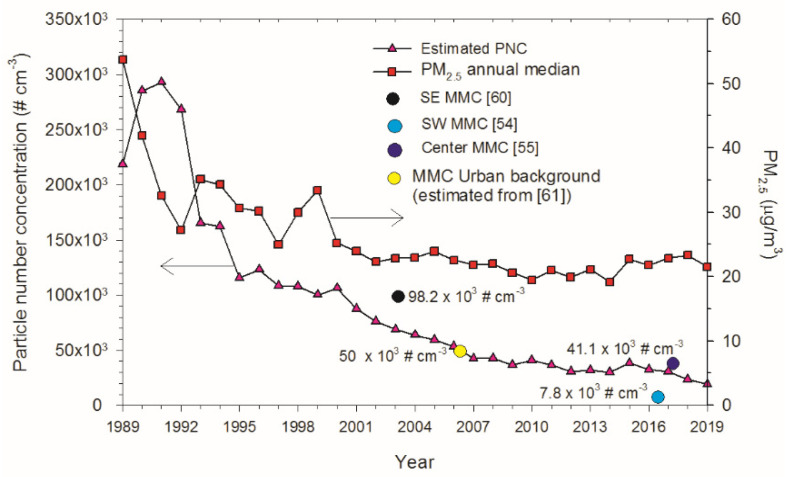
Annual PNC trends estimated from the medians of annual measured levels of CO and estimated (before 2004) and measured (after 2004) PM_2.5_ registered in the MMC from 1989 to 2019. Source of PM_2.5_ data was: http://www.aire.cdmx.gob.mx/default.php# (accessed on 4 June 2022).

UFPM and NPs have a very low mass compared with larger diameter particles; therefore, the PM fraction <100 nm in MMC was evaluated through measurements of particle number concentration (PNC) [60,61,62,63]. The expected PNC trend in MMC was estimated using CO and PM_2.5_ measurements integrated in a nonlinear regression by our laboratory (CO and PM_2.5_ are tracers of vehicular emissions) [46]. The estimated PNC for the 1990s was around 300,000 cm^−3^. Given that catalytic converters in cars and unleaded gasoline were not enforced in Mexico until the year 2000, MMC CO and PM_2.5_ levels before 2000 were among the highest levels of criteria pollutants registered in North America [57]. MMC residents born before 2002 were exposed to PNCs in the range of 300,000 cm^−3^ and, given that the PNC trend decreased to the overall average of 44,000 cm^−3^ after 2003, exposures of MMC residents reached the average PNC for 40 urban areas across Asia, North America, Europe and Australia precisely after 2003 [63]. Figure 2 shows the annual PNC trends calculated for MMC from 1989 to 2019. The symbols in the figure correspond to the median PNCs and the dates of measurement reported by Dunn et al. [60]—commercial with median industry and heavy traffic site, size of measured UFPMs between 3–15 nm; Caudillo et al. [53]—residential with low traffic site, size of measured UFPMs between 20–100 nm; Velasco et al. [54]—commercial with moderate to heavy traffic, size of measured UFPMs < 50 nm; Kleinman et al. [61]—PNC urban background estimated from the extrapolation down to surface of the average UFPMs with sizes < 100 nm measured by aircraft across the MMC at an average altitude level of 350 m above surface.

MMC is an example of uncontrolled urban growth and environmental pollution [53,54,55,56,57,58,59,60,61,62,63,64]; the area of MMC is over 2000 km^2^, and it lies in an elevated basin 7400 feet above sea level. MMC has nearly 22 million inhabitants, over 50,000 industries and over 5 million vehicles consuming more than 50 million liters of petroleum fuels per day. In this megacity, MMC motor vehicles release abundant amounts of primary PM_2.5_, elemental carbon, particle-bound polycyclic aromatic hydrocarbons, carbon monoxide, nitrogen oxides and a wide range of air toxins and other toxics, including formaldehyde, acetaldehyde, benzene, toluene and xylenes [55,57,58,59,64]. The high altitude and tropical climate facilitate ozone production all year [59] and contribute to the formation of secondary particulate matter. A review of several MMC short-term pilot studies showed that the existing heavy-duty diesel fleet emits high amounts of UFPM [65]. In addition, measurements of UFPM emissions from light gasoline vehicles at smoke checking workshops have also shown that old vehicles are high PM_0.1_ emitters [65].

In this study, children and adult cohorts included residents in MMC, while subjects from small control cities represented the typical provincial areas where air pollution emissions are minimal. MMC northern industrialized and southern residential zones were represented in our samples. Southern MMC children are exposed to significant concentrations of ozone, secondary tracers (NO_3_^−^) and particles with lipopolysaccharides (PM-LPS), while northern children are exposed to higher concentrations of volatile organic compounds (VOCs) and PM_2.5_ and its constituents: organic and elemental carbon, including polycyclic aromatic hydrocarbons, secondary inorganic aerosols (SO_4_^2−^, NO_3_^−^, NH_4_^+^) and metals (zinc, copper, lead, titanium, manganese, chromium and vanadium) [55,56,62,64]. Across MMC, residents are exposed to volatile organic compounds (VOCs) and polycyclic aromatic hydrocarbons (PAHs) complex mixtures containing over 100 compounds associated with fine particle-bound PAHs [58,64]. These PAHs are abundant in indoor and outdoor air and busy roadways and are associated with frying oils and snacks and a wide range of occupational exposures [64]. MMC residents are also exposed on daily bases to high outdoor concentrations of Hg in PM_2.5_ [62].

### 2.2. CSF and Brain Samples

This prospective study was approved by the review boards and ethics committees at the Universidad del Valle de Mexico, on the 18 November 2020, and the University of Montana, IRB-206R-09, on the 8 December 2016, and IRB 185-20, on the 23 November 2020.

Normal CSF samples were selected from two cohorts admitted for investigation of neurological involvement in the context of a hematological, neoplastic or infectious brain processes: (i) children admitted to the hospital from a clean air city or MMC, with a work-up diagnosis of acute lymphoblastic leukemia (ALL) entering a clinical protocol that included a spinal tap, and (ii) young and older adults admitted to the hospital for a work-up that required a spinal tap with varied diagnoses, including potential neoplastic and infectious CNS involvement. The cohort of young and older adults also included permanent residents in clean environments and lifetime residents in highly polluted MMC. The normal CSF samples studied were destined to be destroyed after the diagnosis of normal CSF in the laboratory. We also examined 19 CSF ALS cases from Dartmouth-Hitchcock Medical Center Cerebral Spinal Fluid Bank for research IRB #29104 and 5 Mexican ALS cases under the Universidad del Valle de Mexico 18 November 2020 IRB.

The autopsy cases with cerebello-medullary cisternal tap included 15 MMC subjects and 5 low pollution control cases; all had complete forensic autopsies, including full neuropathological examination. These autopsies were performed between 2004 and 2008 under a Consejo de la Judicatura del Distrito Federal permit (14571/2003).

#### 2.2.1. CSF Spinal Taps

Spinal tap was performed on the left lateral decubitus from the intervertebral spaces L3-S1 using a standard 22 spinal needle. Spinal taps were performed between 8 and 10 am. CSF was collected dripping in free air in 1 mL aliquot into Nalge Nunc polypropylene CryoTubes. Lumbar puncture samples were collected during non-traumatic, non-complicated procedures. CSF samples were examined immediately to determine haematological, oncological or infectious involvement and then stored at −80 °C and kept frozen until the current analysis. CSF pleocytosis was defined as CSF white blood cell (WBC) counts of ≥7 cells per mm^3^. All CSF samples in this study were read as normal. We performed the hTDP43 total ELISA (Cat. No. 847-0108000107, Roboscreen GmbH, Leipzig, Germany) according to the instructions for use. This ELISA uses two monoclonal antibodies directed at amino acids 79–91 and 259–271.

#### 2.2.2. Pediatric Cohort for the Measurement of TDP-43 in CSF

This work includes CSF data from a pediatric cohort: 92 children from MMC (33F/59M, mean age = 10.27 years, SD = 4.73) and 26 low pollution control children (11F/15M, mean age = 11.5 years, SD = 4.4). The selected children had no previous oncologic and/or hematologic CNS or systemic treatments and their CSF samples were read as normal, with CNS involvement being ruled out at the time of their hospitalization. Children’s clinical inclusion criteria were negative known smoking history and environmental tobacco exposure, lifelong residency in MMC or the control city, residency within a radius of 2.5 miles of one of the representative city monitoring stations, full-term birth and unremarkable clinical histories prior to their admission to the hospital. We specifically excluded children with a history of active participation in team sports with high incidences of head trauma, including soccer. Mothers had unremarkable, full-term pregnancies with uncomplicated vaginal deliveries and took no illicit drugs, including alcohol and tobacco. These children had a history of breast feeding for a minimum of 6 months and were introduced to solid foods after the age of 4 months. Participants were from middle class families, living in single-family homes with no indoor pets, used LP gas for cooking and kitchens were separated from the living and sleeping areas. Low and high pollution-exposed children were matched by age, gender and socioeconomic status.

#### 2.2.3. Adult Cohort for the Measurement of TDP-43 in CSF

We had 19 ALS cases (9F/10M, average age 52.4 ± 14.1 y), of which 14 were from NH and 5 from MMC; 43 normal MMC CSF samples (27M/16F, average age 43.2 ± 15.9 y); and 14 samples from residents in low pollution cities (9M/5F average age 33.1 ± 12.0 y).

#### 2.2.4. Children and Adult Forensic Autopsies for the Measurement of Cisternal CSF TDP-43 and Complete Brain Examination Including Immunohistochemistry for TDP-43

This work included forensic cases from MMC and low pollution controls. A total of 20 subjects (1F, 19M) (mean age 42.3 years, *SD* = 17.9, age range 16 to 83 y) were examined: 15 MMC male residents, average age 41.13 ± 18.9 y, and 5 controls (1F/4M, 46 ± 16 y). The inclusion criteria for this cohort included lifelong residency in MMC or the control cities and a cause of death—i.e., accidents, homicides and suicides—that did not involve the brain directly. Immunohistochemistry was performed using the TDP-43 mab2G10 (847-0102007401, Cat. No. Roboscreen GmbH, Leipzig, Germany, 1:1000). This antibody is a recombinant human TDP-43 directed to the amino acids MTEDELREFFSQYGDVM of the TDP43 protein and able to identify prominent physiological nuclear immunostaining and neuronal pathological cytoplasmic immunoreactivity in neurons and glial cells in TDP-43 proteinopathy cases.

#### 2.2.5. Data Analysis

We first calculated summary statistics of the TDP-43 concentrations, age and gender for the following cohorts: control children, MMC children, ALS patients, MMC adults and control adults. We then compared the means of TDP-43 concentration between the ALS cohort and controls and between the ALS and the MMC group. We performed two-sample t-tests for this purpose. Next, we investigated the role of age, gender and residency on TDP-43 concentrations in all subjects. For this purpose, we considered linear regression analysis. Since the TDP-43 values varied widely, an appropriate mathematical transformation was necessary so that linear regression analysis could be applied and interpreted reasonably. Logarithm transformation was a natural choice. We performed linear regression of the logarithm of TDP-43 concentration for age, gender and residency. Thereafter, we performed linear regression analysis of the logarithm of TDP-43 concentrations for age and gender. The statistical analyses were performed using the statistical software R.

## 3. Results

### 3.1. CSF TDP-43

CSF samples were colorless, with normal opening pressure. Table 1 shows the mean ± SD and 25th, median and 75th percentile results for ALS cases, MMC and lower pollution control children and adult samples.

**Figure 3:**
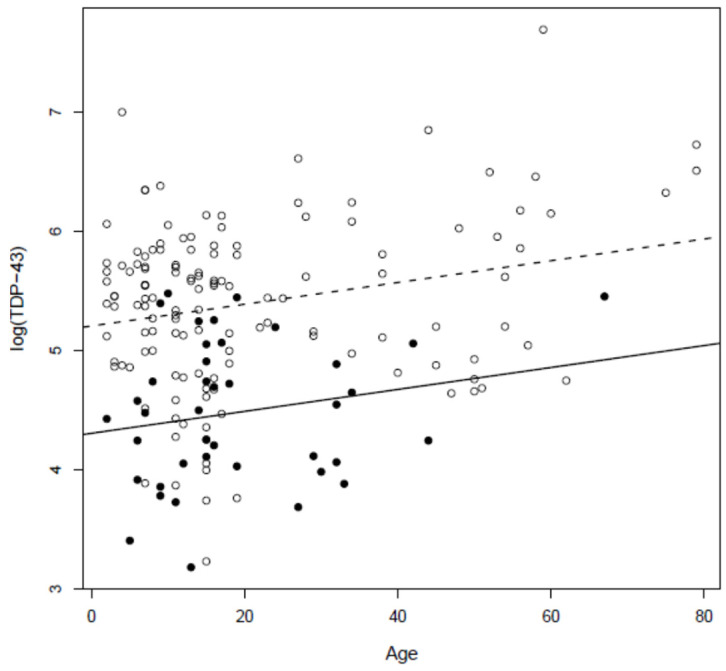
Linear regression of logarithm of TDP-43 CSF concentrations for age and residency (controls and MMC residents). Logarithm of TDP-43 increased significantly with age (*p* = 0.0032). Black circles represent controls and open circles MMC residents.

Mean CSF TDP-43 concentration was significantly higher in ALS versus MMC and non-MMC controls (*p* < 0.0001). The logarithm of TDP-43 increased significantly with age (*p* < 0.0001) and it was higher for MMC residents (*p* < 0.0001). Excluding ALS cases, a log-transformed linear regression of TDP-43 concentration relative to age and residency showed that CSF TDP-43 increases significantly with age (*p* = 0.0032) (Figure 3).

### 3.2. Cisternal CSF TDP-43 and Brain Pathology

The average age of the MMC adult cases was 41.1 ± 18.9 years and that of the controls was 46 ± 16 y. A 16-year-old MMC boy was also studied. Table 2 shows the neuropathological markers in each case and the concentrations of cisternal CSF TDP-43. There was a significant difference between the CSF TDP-43 concentrations in controls without brain pathology versus the six MMC cases showing TDP-43 brain pathology: controls—265.2 ± 132 vs. MMC—572.5 ± 228.5 pg/mL. The mean MMC TDP-43 cisternal concentration was 350.8 ± 247 pg/mL.

Remarkably, concentrations of TDP-43 in cisternal CSF with an average of 572 ± 208 pg/mL forecasted cortical and subcortical TDP-43 pathology, while averages of 232 ± 130 pg/mL were associated with negative TDP-43 brain pathology in MMC residents and controls.

Table 3 shows the TDP-43 immunoreactivity in the 6 MMC cases with TDP-43-related brain pathology and targeted anatomical locations. A total of 25 cortical and subcortical regions were immunoassayed with a TDP-43 antibody and/or counter-stained with hematoxylin. Regions were evaluated for: (1) complete loss of nuclear TDP-43 expression; (2) dash immunoreactive (IR) particles in the vicinity of neuronal nuclei; (3) skein-like tangles in neurons; (4) glial pathology, including oligodendroglia coil tangles. We sectioned the entire brainstem up to cervical levels C1–C2 and included the olfactory bulb; frontal, temporal and parietal cortices; hippocampus, caudate and putamen; substantia nigrae; locus coeruleus; cranial nerve nuclei; trigeminal ganglia; and cervical sections C1 and C2 in our immunohistochemistry studies.

**Figure 4:**
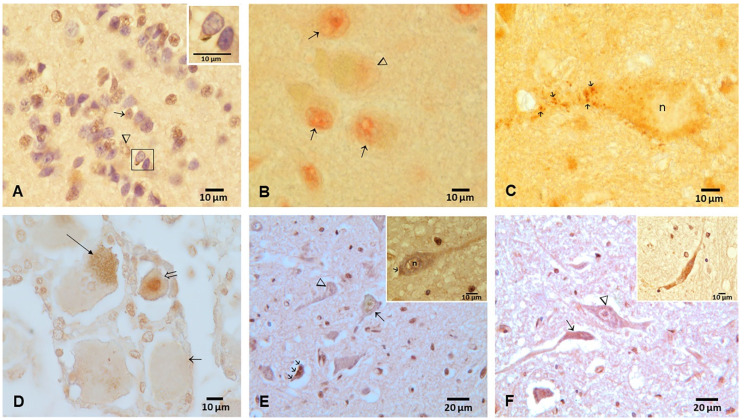
Olfactory bulb, trigeminal ganglia and brainstem motor nuclei immunohistochemistry for TDP-43 in young Metropolitan Mexico City residents. (**A**) Olfactory bulb granule cells in a 25 y old male showing complete loss of nuclear TDP-43 IR (arrowhead) alternating with nuclear TDP-43 (short arrow), the presence of short tangles and loss of nuclear expression (square and insert, the arrow points to a short TDP-43 positive tangle in a granule neuron). TDP-43 and DAB (brown staining). (**B**) Oculomotor nucleus (III cranial nerve) in the 16 y old showing a neuron (arrowhead) with complete loss of nuclear IR surrounded by three neurons with strongly stained red nuclei (short arrows). (**C**) 40 y old male trigeminal spinal neurons with negative nuclei and cytoplasmic dash-like IR, mostly in the axonal region (short arrows). (**D**) 45 y old male trigeminal ganglia neurons with negative nuclei and cytoplasmic positivity (long arrow), alternating with negative nuclear IR (short arrow) and strong nuclear TDP-43 IR (arrowhead). (**E**) 16 y male vagal neurons with negative nuclei (arrowhead), neurons with weak nuclear IR (long arrow) and neurons with intense cytoplasmic inclusions (three short arrows). DAB, brown product. Insert: Vagal neuron with a negative nucleus (n) and positive cytoplasmic IR (short arrow). (**F**) 16 y old male hypoglossal neurons with negative IR nuclei (arrowhead) alongside a neuron with intense cytoplasmic IR (arrow). Insert. hypoglossal neuron with intense IR granular material. DAB, brown product.

**Figure 5:**
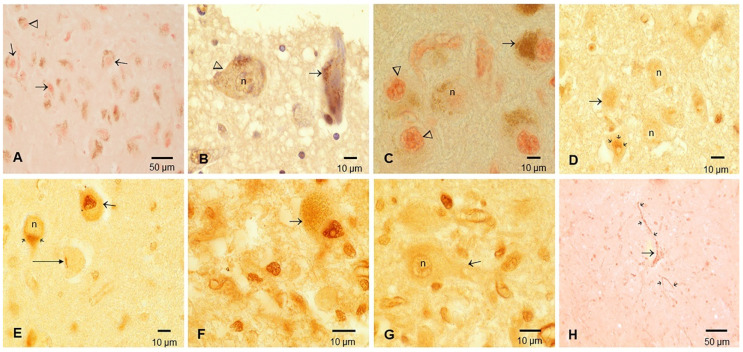
TDP-43 immunohistochemistry in locus coeruleus (LC), substantia nigrae (SN), pons and medulla. (**A**) Locus coeruleus in a 24 y old showing the spectrum of TDP-43 nuclear pathology from positive nuclear staining (short arrows) to negative nuclear IR (arrowhead). (**B**) Sixteen y old male with LC neurons showing negative nuclei and scanty dash-like IR (arrowhead), along with negative nuclear IR and prominent granular cytoplasmic IR (arrow). DAB, brown product. (**C**) 42 y old male SN neuron with IR negative nucleus (n) contrasting with IR-positive nuclei (arrowheads) with a few melanin granules and a neuron with abundant neuromelanin and IR-positive nucleus (arrow). Red product. (**D**) Cluster of pontine neurons in a 42 y old with IR-negative nuclei (n), fine granular cytoplasmic IR (arrow) and paranuclear strong IR (three arrows). DAB, brown product. (**E**) 42 y old male inferior olivary complex with unremarkable neurons (short arrow), nuclear IR negativity (n) and strong granular paranuclear IR (two short arrows). One neuron with a short tangle and a negative IR nucleus (long horizontal arrow) is also identified. (**F**) Seventy-five y old male area postrema with large neuron with strong nuclear IR (arrow). (**G**) Same subject as F with area postrema showing a large neuron (arrow) with nuclear (n) IR negativity. (**H**) Forty y old male medullary reticular neuron (long arrow) with nuclear IR negativity and extensive granular IR along axonal and dendritic tree (short arrows).

**Figure 6:**
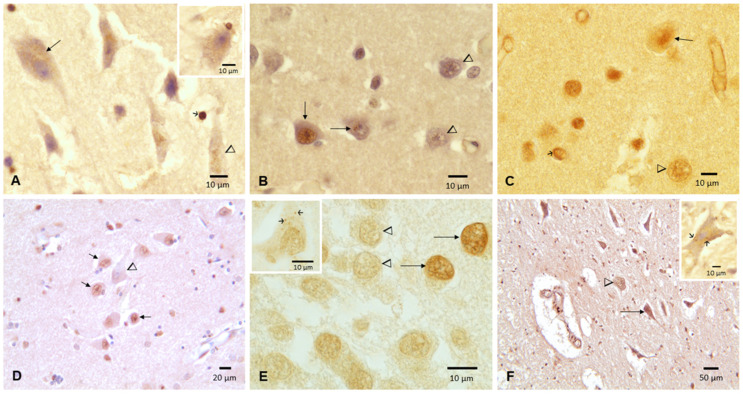
TDP-43 Immunohistochemistry in frontal and temporal cortex, hippocampus and cervical motor neurons. (**A**) Twenty-four y old male showing a frontal pyramidal neuron with negative nuclear IR and dash-like cytoplasmic positive IR (long arrow), a pyramidal neuron with cytoplasmic IR and negative IR nucleus (arrowhead) and a glial cell with strong nuclear IR (short arrow). Insert shows a coil tangle in an oligodendroglia cell close to a frontal motor neuron with negative IR nucleus and a cytoplasmic IR. DAB counterstained with hematoxylin. (**B**) Twenty-five y old male temporal cortex showing unremarkable neurons with strong nuclear IR (long vertical arrow) contrasting with nuclear negative IR (arrowheads) and one neuron with negative nuclear IR and a small cluster of IR positive granules (horizontal arrow). (**C**) Forty-two y old male temporal cortex with normal neurons with strong nuclear IR (long arrow), neuronal negative nuclear IR (arrowhead) and coil tangle in an oligodendroglia (short arrow). (**D**) Seventy-five y old hippocampus CA2 neurons with nuclear IR (short arrows) and negative nuclear IR (arrowhead). (**E**) Same case as (**D**), dentate hippocampal gyrus neurons with strong nuclear IR (arrows) contrasting with nuclear negative IR (arrowheads). Insert is a hippocampal neuron in CA3 in the same subject showing a negative nuclear IR and a few positive cytoplasmic granules. (**F**) Sixteen y old male cervical (C2) anterior horn motor neuron with negative nuclei (arrowhead) and one neuron with cytoplasmic IR (long arrow). Insert: This cervical motor neuron in the same child exhibits a negative IR nucleus and a few positive IR cytoplasmic granules.

Representative immunohistochemistry photographs are shown in Figure 4, Figure 5 and Figure 6. Positive TDP-43 pathology in the six MMC autopsy cases was characterized by complete loss of nuclear TDP-43 expression and/or dash-like IR in the vicinity of neuronal nuclei (Figure 4). We documented olfactory bulb granule cell short tangles in young adults (Figure 4A).

The pathology was mostly seen in brainstem levels, including the medulla and pons, substantia nigrae and locus coeruleus, and in relation to the mesencephalic reticular formation, ventral tegmental area-parabrachial pigmented nucleus complex and caudal-rostral linear nucleus of the raphe (Figure 5).

The cortical and hippocampal pathology was characterized by loss of nuclear TDP-43 expression and/or dash-like IR cytoplasmic neurons. The 24 and 25 y old subjects showed negative IR nuclei in frontal and temporal neurons, cytoplasmic IR and oligodendroglia tangles (Figure 6A,B), while the 16 y old boy exhibited cervical motor neurons with IR negativity and cytoplasmic IR (Figure 6F).

## 4. Discussion

Intracellular accumulation of abnormal misfolded proteins is the critical neuropathological feature of ALS, FTD, Alzheimer’s and Parkinson’s diseases and exposure to particulate matter air pollution has been described as a strong risk factor for Metropolitan Mexico City (MMC) young residents [1,2,3,4]. Mexican urbanites exposed to complex mixtures of air pollutants, including PM_2.5_ above current EPA US standards and highly toxic UFPM have CSF TDP-43 concentrations exponentially increasing with age. TDP-43 is the target protein associated with ALS and fronto-temporal dementia (FTD) [39,40,41,42,43,44,66,67,68]. Remarkably, concentrations of TDP-43 in cisternal CSF with averages of 572 ± 208 pg/mL forecasted cortical and subcortical TDP-43 pathology, while averages of 232 ± 130 pg/mL were associated with no TDP-43 brain pathology in any of the study subjects. Our neuropathology findings in the six MMC individuals included loss of normal nuclear TDP-43 expression and development of cytoplasmic aggregations in non-motor and motor neuronal groups and involved the olfactory bulb, brainstem and cervical motor neurons. Our previously published MMC cases [2] with TDP-43 pathology involved non-motor nuclei, as described in the work by Pandya [67]. Pandya et al. investigated 79 ALS cases using a network diffusion model (NDM) to define whether a process of focal pathological ”seeding”, followed by structural network-based spread, recapitulated postmortem histopathological staging, and if there was any correlation with the pattern of expression of a panel of genes implicated in ALS across the healthy brain. Remarkably, the critical seed regions for spread within the model were the thalamus, insula, pallidum, putamen and caudate [67]. This information is very relevant to our Mexico City studies, since we have a significant overlap of abnormal neural proteins [1,2], and non-primary motor regions are among the earliest sites of cerebral TDP-43 pathology. Moreover, the involvement of the frontal and temporal cortex and cerebellum in frontotemporal lobe dementia FTLD-TDP patients should be noted, as described in the work of Hasan et al. [68]. Equally relevant to our previous MRI and magnetic NPs quantification in MMC residents’ cerebellum [2,47,69], Hasan et al. identified strong cerebellar involvement [68], which most certainly can be used as early evidence of neurodegeneration in MMC residents [2,4,46,69].

The global connectome architecture of FTD subjects is a relevant issue in early TDP-43 pathology, as it allowed researchers such as Shafiei et al. [70] to identify regional early atrophy; i.e., insula—which was identified as the predominant group epicenter of brain atrophy using data-driven and simulation-based methods—with secondary regions in frontal ventromedial and antero-medial temporal areas [70]. These critical observations [70] associating atrophy patterns in sporadic and genetic bvFTD with global connectome architecture and local transcriptomic vulnerability provide an explanation as to how quadruple pathology could account for overlapping clinical pictures [2,4,45,46,71]. Scarioni and colleagues [72] were able to dissect 20 standard neuropathology regions associated with FTLD-TDP, FTLD-tau and FTLD-fused-in-sarcoma (FTLD-FUS) and stained them for phosphorylated TDP-43, phosphorylated tau, FUS, amyloid-beta and alpha-synuclein. Their results showed TDP-43 pathology in the hippocampal granular layer correlates with hallucinations, CA1 with mania, CA3 with depression and parahippocampal tau with delusions. In the brainstem, the presence of alpha-synuclein co-pathology in the substantia nigra (SN) correlated with disinhibition, tau pathology in the SN with depression and tau pathology in the locus coeruleus with both depression and perseverative/compulsive behavior. The results of Scarioni et al. [72] strongly support that a subcortical TDP-43 burden contributes to configuring the clinical phenotype of FTLD. Recent important publications have described end-stage FTLD with tau or TDP-43 pathology [73,74], emphasizing laminar distributions of lower laminar tau, higher TDP pathology and Fe-rich cortical inflammation in tau vs. TDP cases. We were unable to corroborate such distributions in our cases. Our MMC cases demonstrated significant concentrations of highly reactive, magnetic Fe nanoparticles distributed throughout cortical, olfactory, brainstem and cerebellum regions [2,24,25,46].

Our TDP-43 CSF findings and the previously documented quadruple brain pathology [1,2,3,4] are in keeping with the presence of several abnormal CSF proteins in the seemingly CNS-uncompromised MMC versus low pollution control children [30,31,32,33,34] with low amyloid-β1-42 and BDNF [32,33] and elevated MIF, IL6, IL1ra, IL2 and PrP(C) levels [30].

The abnormal CSF proteins found in MMC children and young adults are very concerning because they are involved in several pathophysiological mechanisms of early neural damage with eventual impacts on cognitive, neurological and psychiatric outcomes [71,75,76].

At the core of our results, we report accumulation of TDP-43 starting at an early age in individuals highly exposed to complex environmental emissions, including UFPM [1,2,3,4]. The TDP-43 pathology observed in young MMC children [2] and the correlation between cisternal TDP-43 levels and the distribution of pathology in children and young adults cannot be dismissed.

Non-fibrillary TDP-43 accumulates in the rough endoplasmic reticulum (RER), as described by Kon and colleagues [77], and the ER is one of the targets of UFPM/NPs in MMC young residents [1,2,24]. To this end, the work of Scarioni and colleagues is key [72]. Their robust data established associations between clinical symptoms and TDP-43 pathology. Strikingly, MMC residents have cellular, subcellular and immunoreactive pathology in precisely the same regions [2,46,69]. Moreover, Andrew and colleagues [78] identified contaminants found to have a strong association with ALS risk in the US, including airborne lead (false discovery rate (FDR) = 0.00077), primarily produced by small aircraft, and polychlorinated biphenyls (PCBs), such as heptachlorobiphenyl (FDR = 3.60E-05) emitted by power plants burning biomass and industrial boilers. This analysis supports neurotoxic airborne metals and PCBs as risk factors for ALS.

Neuropathological information in conjunction with the social brain hypothesis [79] and the application of targeted cognitive behavioral function tests [80] for highly exposed cohorts is a path we could explore, knowing social and criminal transgressions are recorded in bvFTD patients [81]. Mendez discussed precisely this impairment in an innate sense of morality and the predominantly right-hemisphere pathology in frontal (ventromedial, orbitofrontal, inferolateral frontal), anterior temporal (amygdala, temporal pole), limbic (anterior cingulate, amygdala) and insular regions in bvFTD patients [81]. MMC subjects show involvement of these key areas, as explored by brain MRI [69], and social violence in MMC is significant [82]. According to a 2020 report by the Mexico City-based Citizen Council for Public Safety and Criminal Justice, seven out of the ten “most violent” cities in the world were located in Mexico [83]. For the country as a whole, 2019 and 2020 were the most violent on record, with more than 34,000 intentional homicides each year, and the Mexico state—a very polluted particulate matter region—has the highest incidence of violence [83].

The involvement of TDP-43 in the complex reticular formation in MMC residents must be pursued further, given the complexity of the brainstem reticular nuclei and their connections with supra- and infra-cortical structures involving major integration and relay centers coordinating survival functions and key connections between the cerebral cortex, the cerebellum and the spinal cord [84,85,86,87,88,89,90,91,92,93,94,95,96]. We are particularly concerned about the associations between reticular brainstem-impaired connectivity and isolated sleep behavior disorder (iRBD) [88], one of the earliest manifestations of α synucleinopathies, which is present in MMC young adults [46], as well as the higher risk of falls [90,94] in young MMC adults [69,97].

Research regarding TDP-43, including novel nuclear pore articles, muscle TDP-43 involvement and the anatomical targets [39,40,41,42,43,44,45,98,99,100,101,102,103,104,105,106,107,108,109,110,111], must be revisited in the context of potential early involvement in pollution-exposed populations. For example, it could be particularly intriguing to look for nuclear NPs in motor neurons of highly exposed city dwellers [107] and hypothalamic TDP-43 lesions [111].

There is an urgent need for noninvasive, reliable fluid biomarkers to diagnose early TDP-43 pathology to improve the differential diagnosis with overlapping neurodegenerative diseases that undoubtedly exhibit an extreme variability in clinical phenotypes, as described by Virgilio et al., for tau [112]. Since TDP-43 pathology increases linearly with age in older adults with and without dementia [113], the issue of prevention at early ages is certainly crucial. Identifying the individuals at highest risk of MMC TDP-43 pathology is one of our goals, and to the exploration of cognitive behavioral function tests we could add lipid metabolic traits and body complexions causally associated with the risk of FTD, as in the work of Esteban-García et al. [114]. Increased trunk-predicted mass and fat-free mass, as well as higher circulating triglyceride levels, impact differentially on the risk of FTD and ALS [114].

The work of researchers exploring correlations between fluid biomarkers is highly relevant [115,116,117,118,119,120,121,122]. In particular, our interest is focused on plasma biomarkers to help distinguish between MCI + AD and controls and between FTD and progressive supranuclear palsy (PSP); i.e., P-tau181 [122]—the positive correlation between CSF and plasma values for NfL (*p* < 0.0001), with NfL values higher for all phenotypes of symptomatic FTD-ALS spectrum (FAS) patients compared to primary psychiatric disorders (PPD) [121].

Since, our MMC cases have AD hallmarks, and given that cognition, gait and balance; auditory-evoked brainstem potentials; and brain MRI changes are striking in the young MMC residents [1,2,16,46,69,97,123,124,125]—we are obligated to include AD biomarkers [118,126,127]. Since CSF samples continue to be the gold standard of neurodegenerative biomarkers, exploration of normal CSF samples in the hospital setting will continue to be an excellent source of samples. Thus, multi-marker synaptic proteins [128], CSF phosphorylated-tau levels (associated with cerebral tau burden in FTLD) and TDP-43 may help to define, in autopsy materials, the burden of TDP-43 pathology and identify risk factors [129,130]. Forensic autopsy-confirmed neurodegenerative biomarkers and neuropathology hallmarks are crucial to define the pathology burden in a population at large.

There were advantages and limitations to this study. A major advantage was the multidisciplinary collaborators and the efforts made to exchange viewpoints regarding the importance of early clinical detection of young individuals at risk, key in understanding the impact of our findings in future multidisciplinary studies. The current particle number concentration (PNC) in MMC is crucial environmental information for the responsible authorities. Residents continue to be exposed to an average of 44,000 particles per cm^−3^ [63], with all the health-associated risks. There is a need to define the presence of the major ALS mutations, including the C9orf72 expansion mutations, SOD1 and TARDBP gene mutations, in MMC residents, and this study further highlights the need to consider early TDP-43 pathological changes as an opportunity to explore pathomechanistic avenues where we could intervene to halt neurodegenerative processes.

## 5. Concluding Remarks

1. Particulate matter exposures—specifically, UFPM/NP sustained exposures from utero—are likely key in aberrant neural protein pathology. The CSF TDP-43 results identify logarithmic increases related to age across young megacity urbanites, crucial information in view of the 18% TDP-43 pathology reported in 202 forensic MMC autopsies aged 27.29 ± 11.8 y and the overlap of aberrant hyperphosphorylated tau, beta amyloid, α synuclein and TDP-43. These data are striking in view of the work of Karanth et al. [71] and Carlos et al. [113]. Karanth and coworkers pointed out that quadruple misfolded proteins, including tau neurofibrillary tangles, amyloid-β [Aβ], α-synuclein and TDP-43, in the same brain are relatively common in aging. Moreover, dementia frequency was highest among those with quadruple misfolded proteins [71]. Carlos et al. [113] made a calculation of the frequency and distribution of TDP-43 pathology in 1072 cases, average age 87 years, with AD and TDP-43 pathology and antemortem cognitive studies, including 58% with dementia, 15% with mild cognitive impairment and 27% who were cognitively intact. Carlos and colleagues showed a linear increase in TDP-43 pathology with age: 30% of subjects aged 70 had TDP-43 pathology, 42% by age 80 y and 49% by age 90. Strikingly, these cases were white residents in a low-polluted area, reflecting that elderly TDP-43 increases linearly, notwithstanding the low cumulative exposures to environmental air pollutants, in sharp contrast with our logarithmic increases in pediatric and young adults with high UFPM/NP exposures already exhibiting quadruple misfolded protein pathology.
2. We identified a significant relationship between cisternal CSF TDP-43, an average of 572 ± 208 pg/mL, and TDP-43 brain pathology. This is particularly serious for highly exposed children, as with the 16 y old boy (1013 pg/mL) who had extensive nonmotor and motor TDP43 pathology. As toxicologists working with forensic colleagues, we suggest that forensic cases are very helpful to explore the extent of brain pathology in a population at large, and taking a cisternal sample is simple and quick.
3. Defining early markers of the quadruple aberrant neurodegenerative diseases, including TDP-43 pathology, ought to be the core of our future efforts. MMC residents are showing early clinical symptomatology—including gait and balance alterations, cognitive deficits and MRI volumetric cortical and subcortical abnormalities, all of which may help identify young subjects at higher risk.
4. Exposed children and young adults in highly polluted areas need early neuroprotection and multidisciplinary prevention efforts. Control of combustion and friction UFPM sources and engineered NPs (food products, cosmetics, toothpaste, sun protectors, surface disinfectants, paints, e-waste) is becoming increasingly important and urgent to diminish the human and economic costs of a global neurodegenerative epidemic.
5. UFPM/NP exposure should be included in any assessment of the neurodegenerative risk profile of exposed individuals. No matter the portal of entry, chronic delivery of exogenous particles to the brain induces oxidative stress and neuroinflammation.
6. We have described the overlap of multiple neurodegenerative pathologies; the presence of anthropogenic UFPM in fetal brains; and the early development of AD, PD and TDP-43 pathology, along with their progression and their neuropsychiatric consequences: this body of knowledge resulting from multidisciplinary studies cannot be disregarded by those concerned with public health.
7. We urgently need to practice preventive medicine and develop tools to identify children at risk in order to implement neuroprotective strategies. As neurotoxicologists, we ought to define the mechanistic pathways involving complex NPs containing metalloids, metals, organic compounds, plastics, etc., that can cause extensive brain pathology. As physicians, our focus should be protecting the brains of our future citizens and our younger generations, identifying neurotoxic emission sources and being active players in multidisciplinary teams to prevent, ameliorate or halt neurodegenerative diseases.

## Figures and Tables

**Table 1:** CSF TDP-43 ELISA results in pg/mL for control and MMC children, ALS patients and adult MMC and low pollution controls. In each cell, the first row shows the mean ± SD and the second row the 25th percentile, median and 75th percentile.

CSF Samples	Age and Gender	TDP-43 pg/mL
Control children *n*: 26	11.5 ± 4.4 y (8.25, 12.50, 15.00) y 11F/15M	102 ± 59 (58.14, 88.65, 129.77)
MMC children *n*: 92	10.27 ± 4.7 y (7.00, 11.00, 15.00) y 33F/59M	239 ± 152 (130.28, 229.39, 299.47)
ALS patients *n*: 19	52.4 ± 14.1 y (56.50, 61.00, 64.00) y 9F/10M	902 ± 269 (683.30, 906.07, 1085.79)
MMC adults *n*: 43	43.2 ± 15.9 y (28.50, 45.00, 54.00) y 16F/27M	373 ± 358 (159.86, 275.10, 473.55)
Control adults *n*: 14	33.14 ± 12.0 y (27.50, 32.00, 33.75) y 5F/9M	108 ± 67 (56.49, 81.75, 150.75)

**Table 2:** Autopsy cases with cisternal CSF TDP-43 and complete neuropathological examination using H&E, PHF-tau8 phosphorylated at Ser199-202-Thr205 and α-synuclein phosphorylated at Ser-129, LB509 and TDP-43 mab2G10.

ID	Age	Gender	APOE	AD pτ *	AD Aβ **	SN pτ §	SN αS §§	TDP-43 Brain ‡	TDP-43 Cisternal pg/mL
1	16	1	0	2	2	1	0	1	1013
2	21	1	0	5	3	0	0	0	392
3	24	1	0	2	2	1	1	1	375
4	25	1	0	1	2	0	0	1	565
5	27	1	0	2	2	1	1	0	218
6	37	0	0	5	4	1	1	0	42
7	38	1	0	4	3	1	0	0	306
8	39	1	0	3	2	1	1	0	167
9	40	1	0	4	3	1	1	1	562
10	42	1	0	3	2	1	0	1	496
11	47	1	0	3	2	0	0	0	194
12	48	1	0	4	3	1	1	0	293
13	55	1	0	5	3	0	1	0	150
14	75	0	0	4	3	1	1	1	424
15	83	0	0	4	3	1	0	0	66
1 CTL	29	0	0	0	0	0	0	0	364
2 CTL	34	1	0	0	0	0	0	0	191
3CTL	43	1	0	0	0	0	0	0	435
4 CTL	56	1	0	0	0	0	0	0	226
5 CTL	68	1	0	0	0	0	0	0	110

* AD staging—pτ stage: 0 = absent; 1 = pre-tangle stages a–c; 2 = pre-tangle stages 1a and 1b; 3 = NFT stages I and II; 4 = NFT stages III and IV; 5 = NFT stages V and VI. ** AD staging—Aβ phase: 0 = absent; 1 = basal temporal neocortex; 2 = all cerebral cortex; 3 = subcortical portions—forebrain; 4 = mesencephalic components; 5 = reticular formation and cerebellum. § Substantia nigrae pτ was evaluated as pre-tangles, positive neurites and tangles using the PHF-tau8 phosphorylated at Ser199-202-Thr205 (Innogenetics, Belgium, AT-8 1:1000). §§ Substantia nigrae α-S was evaluated as neuronal immunoreactive (IR) aggregates in the somato-dendritic compartment, cytoplasmic inclusions, core-halo Lewy bodies and dystrophic neurites (Lewy neurites) using α-synuclein phosphorylated at Ser-129, LB509 (In Vitrogen, Carlsbad, CA 1:1000). ‡ Brain TDP-43 was evaluated as dash-like IR particles in the vicinity of the cell nucleus, with or without complete loss of nuclear TDP-43 expression and somatic skein inclusions and glial pathology using mab2G10 (Roboscreen GmbH, Leipzig, Germany 1:1000).

**Table 3:** Neuropathology findings in the six MMC males with cisternal CSF TDP-43 data and TDP-43 neuropathology. Numbers represent: (1) complete loss of nuclear TDP-43 expression; (2) dash-like immunoreactive (IR) particles in the vicinity of neuronal nuclei; (3) skein-like tangles in neurons; (4) glial pathology, including oligodendroglia coil tangles. * Cranial nerve nuclei.

Anatomical Areas	16 1013 pg/mL	24 375 pg/mL	25 565 pg/mL	40 562 pg/mL	42 496 pg/mL	75 424 pg/mL
Frontal motor	1	1	1	1	1, 2	1, 2
Frontal non-motor	1	1	1	1	1	1
Parietal	1	0	0	1	1	1
Temporal	1	1	0	1	1, 2	1
Hippocampus	1	1	1, 2, 4	1, 2	1, 2	1, 2, 4
Caudate	1	0	1	0	0	0
Putamen	0	0	1	0	0	1
Globus pallidus	0	0	1	0	1	0
XII *	1, 2	1, 2	1	1	1,2	1
X *	1	1, 2	1	1	1	1
IX *	0	0	1	1	0	0
VIII *	1	1	1	0	0	1
VII *	1	0	1, 2	1,2	1	1
VI *	0	1	1	0	0	0
V *	1, 2	1	1, 2	1	1	1
IV *	1	1	1	0	1	1
III *	1	1	1, 2	1	1	1
II *	0	0	0	0	0	0
I *	1	1	1, 2, 3, 4	1	1, 2	1
Substantia nigrae pc	1, 2	1, 2	1	1, 2	1, 2	0
Locus coeruleus	1, 2	1	1	1, 2	1, 2	1, 2
Pons neurons	1, 2, 4	1, 2, 4	1, 2	1, 2	1, 2	1, 2
Mesencephalic reticular formation	1, 2	1	1, 2	1, 2	1, 2	1
Area postrema	1	1	1, 2	1, 2	1	1
Trigeminal ganglia	0	1	1	1, 2	1, 2	1, 2
Inferior olivary complex	1	1	1	1	1	1
Cervical, anterior horn	1	1, 2	1, 2	1	1	1

## Data Availability

All data necessary to understand and assess the conclusions of this study are available in the main text.
